# Supplementary material for: Content-rich biological network constructed by mining PubMed abstracts
Source: BMC Bioinformatics. 2004 Oct 8;5:147. doi: 10.1186/1471-2105-5-147 (PMC528731; doi:10.1186/1471-2105-5-147)
Supplement: Additional File 2 — The original results of the above study (non-essential files are deleted to keep the file size under the limit set by BMC bioinformatics). [file 1471-2105-5-147-S2.bz2 › chilibotAdditionalFile2/dip10/769ID10633096E18160/html/FECI_FECR.html]

 


 **FECI** and **FECR** 
  
Found 19 abstracts in PubMed, retrieved 10.  
 

 What does Google say? 
 PDF only 
| .edu only 

---

**Interactive relationship** (e.g. stimulation, inhibition, etc)

**Neutral relationship**- Surface signaling in ferric citrate transport gene induction interaction of the FecA,  **FecR** , and  **FecI**  regulatory proteins.  Ref: 10633096 J Bacteriol, 2000
- The results demonstrate that region 4 of  **FecI**  specifically binds  **FecR** .  Ref: 12057967 J Bacteriol, 2002
- FecA 1 79 interacts with  **FecR**  101 317 and  **FecR**  1 85 interacts with  **FecI**  1 173.  Ref: 10633096 J Bacteriol, 2000
- The data reveal properties of the  **FecI**  ECF factor distinct from those of sigma 70 and further support the novel transcription initiation model in which the cytoplasmic portion of  **FecR**  is important for  **FecI**  activity.  Ref: 11114913 J Bacteriol, 2001
- Interaction of N proximal regions of predicted  **FecR**  homologs with region 4 of predicted  **FecI**  homologs of Pseudomonas aeruginosa was demonstrated.  Ref: 12057967 J Bacteriol, 2002
- no interactions occurred between heterologous combinations of the P. aeruginosa proteins and between a P. aeruginosa  **FecI**  homolog and E. coli  **FecR** .  Ref: 12057967 J Bacteriol, 2002
- In in vitro binding assays,  **FecR**  that was His tagged at the N terminus His 10  **FecR**  and bound to a Ni nitrilotriacetic acid agarose column was able to retain FecA, and  **FecR**  that was His tagged at the C terminus  **FecR**  His 6 retained  **FecI**  on the column.  Ref: 10633096 J Bacteriol, 2000
- These data clearly support a model that proposes interaction of the periplasmic N terminus of FecA with the periplasmic C terminal portion of  **FecR**  and interaction of the cytoplasmic N terminus of  **FecR**  with  **FecI** , which results in  **FecI**  activation.  Ref: 10633096 J Bacteriol, 2000
- The cytoplasmic portion of the  **FecR**  mutant proteins,  **FecR**  1 85, did not interact with wild type  **FecI** , in contrast to wild type  **FecR**  1 85, which induced  **FecI**  mediated fecB transport gene transcription.  Ref: 11114913 J Bacteriol, 2001
- An in vivo deletion analysis using a bacterial two hybrid system assigned the interaction of the  **FecR**  and  **FecI**  proteins to the cytoplasmic portion of the  **FecR**  transmembrane protein and region 4 of  **FecI** .  Ref: 12057967 J Bacteriol, 2002
- Functional interaction of region 4 of the extracytoplasmic function sigma factor  **FecI**  with the cytoplasmic portion of the  **FecR**  transmembrane protein of the Escherichia coli ferric citrate transport system.  Ref: 12057967 J Bacteriol, 2002
- Two missense mutations in region 2.1 of  **FecI** , S15A and H20E, partially restored induction of ferric citrate transport gene induction of the  **fecR**  mutants by ferric citrate.  Ref: 11114913 J Bacteriol, 2001
- **FecR**  then transmits the signal across the cytoplasmic membrane into the cytoplasm and activates the  **FecI**  sigma factor, which binds to the RNA polymerase core enzyme and directs the RNA polymerase to the promoter upstream of the fecABCDE transport genes to initiate transcription.  Ref: 12732308 Curr Opin Microbiol, 2003
- **FecR**  receives the signal in the periplasm from the outer membrane protein FecA loaded with ferric citrate, transmits the signal across the cytoplasmic membrane, and converts  **FecI**  in the cytoplasm to an active sigma factor.  Ref: 12813067 J Bacteriol, 2003
- Interaction of  **FecR**  with  **FecI**  was demonstrated by showing that isolated  **FecR**  inhibited degradation of  **FecI**  by trypsin.  Ref: 12618442 J Bacteriol, 2003
- Control of the ferric citrate transport system of Escherichia coli mutations in region 2.1 of the  **FecI**  extracytoplasmic function sigma factor suppress mutations in the  **FecR**  transmembrane regulatory protein.  Ref: 11114913 J Bacteriol, 2001
- In addition, missense mutations in the  **fecI**  promoter region resulted in a twofold increased transcription in  **fecR**  wild type cells and a partial restoration of fec transport gene transcription in the  **fecR**  mutants.  Ref: 11114913 J Bacteriol, 2001
- Binding of  **FecI**  to beta and beta 1 313 was enhanced by  **FecR**  1 85, which represents the cytoplasmic portion of the  **FecR**  protein that transmits the inducing signal across the cytoplasmic membrane.  Ref: 12618442 J Bacteriol, 2003

**Non-interactive relationship** (e.g. studied together, co-existance, homology, etc.)

- The in vivo assay involved the determination of the FecA,  **FecR** , and  **FecI**  interacting domains with the bacterial two hybrid Lex based system.  Ref: 10633096 J Bacteriol, 2000
- Missense mutations randomly generated by PCR were localized to region 4 of  **FecI** , and the mutants were impaired with regard to the interaction of  **FecR**  with  **FecI**  and fecB lacZ transcription.  Ref: 12057967 J Bacteriol, 2002
- the residual activity of mutated  **FecI**  in the absence of  **FecR**  was not higher than that of wild type  **FecI** .  Ref: 11114913 J Bacteriol, 2001
- We propose that  **FecR**  spans the cytoplasmic membrane once, interacts in the periplasm with its C terminal region with FecA occupied by ferric citrate, and transmits the information through the cytoplasmic membrane into the cytoplasm, where it converts  **FecI**  into an active sigma factor.  Ref: 9573190 J Bacteriol, 1998
- This binding elicits a signal that is transmitted by  **FecR**  across the cytoplasmic membrane into the cytoplasm, where the sigma factor  **FecI**  directs the RNA polymerase to the promoter upstream of the fecABCDE genes.  Ref: 12057967 J Bacteriol, 2002
- Additional ferric citrate specific regulatory proteins are  **FecR**  in the cytoplasmic membrane and the  **FecI**  sigma factor in the cytoplasm.  Ref: 9573190 J Bacteriol, 1998
- Binding of ferric citrate to the outer membrane protein FecA initiates a signal that is transmitted by  **FecR**  across the cytoplasmic membrane into the cytoplasm where  **FecI** , the sigma factor, is activated.  Ref: 10633096 J Bacteriol, 2000
- Bound ferric citrate does not have to be transported but initiates a signal that is transmitted by FecA across the outer membrane and by  **FecR**  across the cytoplasmic membrane into the cytoplasm, where the  **FecI**  extracytoplasmic function ECF sigma factor becomes active.  Ref: 11114913 J Bacteriol, 2001
- Therefore, transcription of the fec transport genes is subjected to double control cells first detect iron deficiency and respond by synthesis of the regulatory proteins  **FecI**  and  **FecR** , which initiate transcription of the fec transport genes, provided ferric citrate is available.  Ref: 12732308 Curr Opin Microbiol, 2003
- By analogy with the  **FecI**   **FecR**  FecA and PupI PupR PupB systems, our data suggest that bfrZ expression is inducible by binding of the cognate ligand to BfrZ and transduction of a signal through the envelope.  Ref: 11292812 J Bacteriol, 2001
- Three specific proteins are involved FecA in the outer membrane,  **FecR**  in the cytoplasmic membrane, and  **FecI**  in the cytoplasm.  Ref: 12732308 Curr Opin Microbiol, 2003
- In 11 cases, a  **fecR**  like gene was found adjacent to the  **fecI**  like gene and, in 10 cases, a gene encoding an iron receptor lies in the vicinity of the  **fecI**   **fecR**  cluster.  Ref: 12534467 Environ Microbiol, 2002
- Five  **fecI**   **fecR**  iron receptor gene clusters from P. putida were also identified in the human pathogen Pseudomonas aeruginosa.  Ref: 12534467 Environ Microbiol, 2002
- Analysis of the genetic region upstream of the bhu cluster identified open reading frames predicted to encode homologs of the Escherichia coli ferric citrate uptake regulators  **FecI**  and  **FecR** .  Ref: 11418569 J Bacteriol, 2001
